# Day surgery in children: 15-year analysis of unplanned admissions at a Japanese tertiary children’s hospital

**DOI:** 10.1007/s00540-024-03445-y

**Published:** 2024-12-21

**Authors:** Aya Sueda, Tetsuro Kagawa, Taiki Kojima

**Affiliations:** 1 Department of Anesthesiology, Hyogo Prefectural Kobe Children’s Hospital, Kobe, Hyogo Japan; 2 Department of Anesthesiology, Aichi Children’s Health and Medical Center, 7-426 Morioka, Obu, Aichi 474-8710 Japan; 3 Department of Comprehensive Pediatric Medicine, Nagoya University Graduate School of Medicine, Nagoya, Aichi Japan

**Keywords:** Ambulatory surgical procedures, Child, Postoperative complications, General anesthesia, Patient readmission

## Abstract

**Purpose:**

Unplanned hospital admission following pediatric day surgery is a crucial quality indicator. This study examined the incidence, related risks, interventions, and outcomes of unplanned hospital admission following pediatric day surgery among children in Japan.

**Methods:**

This single-center, retrospective study analyzed data of 14,529 pediatric patients under the age of 18 years who underwent day surgery between August 2007 and December 2022. Unplanned hospital admission was defined as an overnight hospital stay that was not planned preoperatively, including patients who returned to the emergency department and required admission within 24 h of discharge. Reasons for unplanned hospital admission and interventions were categorized, and risk factors were identified using logistic regression.

**Results:**

The incidence of unplanned hospital admission was 0.19%. The most common reasons for unplanned hospital admission were anesthetic-related, particularly postoperative nausea and vomiting (36%), which was managed primarily with intravenous fluids (36%) and antiemetic medications (21%). Medical and surgical factors were next most common. Logistic regression identified longer operation time (adjusted Odds ratio 1.03; 95% confidence interval [1.01, 1.04]; *P* < 0.001) and exit from the operating room after 15:00 (adjusted Odds ratio 29.3; 95% confidence interval [7.09, 121]; *P* < 0.001) as significant risk factors for unplanned hospital admission.

**Conclusion:**

Unplanned hospital admission was most commonly anesthetic-related and was managed with intravenous fluids and antiemetic medications. Longer operation time and later exit from the operating room were significant risk factors. These findings can guide targeted strategies to further reduce unplanned hospital admission and improve pediatric day surgery quality.

## Introduction

Day surgery, also known as ambulatory or outpatient surgery, is a common surgical approach whereby patients are discharged on the same day as their surgery without an overnight hospital stay [1–3]. It has numerous benefits, including reduced health care expenditure, shorter recovery time, and improved patient satisfaction [1, 3]. Day surgery has a particularly important role in pediatric medicine because it allows children to return to their normal routine and daily activities as soon as possible, minimizing disruption to their lives [1, 3].

Unplanned hospital admission (UHA) after day surgery is an important outcome that reflects patient safety as well as the quality of day surgery and anesthesia [4]. Failure to discharge home on the same day has a negative impact on both patients and their families and health care costs [3]. Therefore, investigating the incidence of and risk factors for UHA after day surgery would contribute to safer planning of anesthesia in high-risk patients.

The data on frequency of UHA following pediatric day surgery derive largely from the North American and European literature and indicate rates in the range of 0.7–2.7% [5–10]. Previous studies in the pediatric population have identified several patient characteristics (e.g., age < 2 years, American Society of Anesthesiologists Physical Status [ASA-PS] score 3 or 4, obstructive sleep apnea [OSA]) and features of surgery (e.g., operation time >1 h and otolaryngology surgery) that could be risk factors for UHA after day surgery [5, 7]. However, while previous research has examined the incidence of UHA following pediatric day surgery, emergency department visits for complications necessitating readmission have only been investigated in a subset of studies. Furthermore, considering the diverse characteristics of patients who experience UHA following day surgery, it is reasonable to anticipate variations in their clinical course and outcomes depending on the reasons for admission. Notably, previous research has not specifically explored interventions associated with UHA. Therefore, further investigation is warranted given this gap in the literature and the limited data specific to the Japanese population [11].

This study examined epidemiological data on the incidence, related risks, interventions, and outcomes of UHA following day surgery among children in Japan.

## Methods

### Ethical considerations

This study was approved by the Institutional Ethics Committee (approval no. R5-51, July 26, 2023) and was conducted in accordance with the principles of the 1964 Declaration of Helsinki and its later amendments. The local ethics committee decided that the need for written consent could be waived, with consent secured via the opt-out route.

### Study design and setting

The study had a single-center, retrospective, cross-sectional design and analyzed data in the electronic medical records held at a tertiary children’s hospital in Japan. Our institution provides comprehensive medical services, including prenatal care for newborns, children, adolescents, and pregnant mothers, serving approximately 190,000 individual inpatients and outpatients annually. The hospital has 290 beds and performs approximately 4200 general anesthesia procedures annually. All patients scheduled for inpatient or outpatient surgery are examined by an anesthesiologist on the day before surgery. Approximately 20–30% of cases are day surgeries, consisting mainly of general procedures as well as otolaryngologic, urologic, and plastic surgery procedures. The main procedures performed as day surgery are inguinal hernia repair, endoscopy, myringotomy with ear tube insertion, orchiopexy, circumcision, and excision of subcutaneous tumors.

## Patients

The study included pediatric patients under the age of 18 years who underwent planned day surgery under general anesthesia at our hospital between August 2007 and December 2022. Procedures not performed under general anesthesia and cases involving emergency or inpatient surgeries were excluded.

### Indications for day surgery

Eligibility for day surgery was determined based on several criteria:

1. Guardian-related conditions:
  within 2 h travel from the hospital;
  understanding of fasting requirements and post-operative care;
  availability for phone follow-up or revisit if necessary.
2. Patient-related conditions:
  age: over 60 weeks post-conception;
  free from recent respiratory infections;
  adequate development and weight gain;
  no severe respiratory issues or difficult airway;
  stable cardiac conditions without pulmonary hypertension;
  well-controlled neurologic conditions;
  no history of malignant hyperthermia.
3. Surgery-related conditions:
  short duration (generally within 1 h);
  minimal expected blood loss;
  manageable post-operative pain with oral medication.

Our stringent selection process involves a thorough pre-operative assessment by both the surgical team and the anesthesiologists. The process begins with initial screening by the surgical team based on the patient’s medical history and planned procedure, followed by a review of medical records and consultations with relevant specialists if needed. A comprehensive pre-anesthetic evaluation is then conducted by a board-certified anesthesiologist, typically the day before surgery, who makes the final decision regarding the patient’s suitability for day surgery. Patients must meet all eligibility criteria to proceed, and if any concerns arise during this assessment, there is an option to convert the case to an inpatient procedure.

### Discharge criteria

A board-certified anesthesiologist determined discharge eligibility using a 10-point scoring system evaluating consciousness, ability to tolerate oral intake, pain control, motor function, nausea and vomiting, respiratory status, circulatory stability, bleeding, body temperature, and time since the last administration of opioids. Patients meeting all 10 criteria were cleared for discharge; those with 9 or fewer points could be discharged at the anesthesiologist’s discretion.

### Collection of data

We retrospectively extracted the following information from the electronic medical records system (Hope EGMAIN-GX^®^, Fujitsu): patient demographics, including age, sex, height, and weight; clinical characteristics, including ASA-PS score, preoperative OSA screening data based on documented snoring and apnea, type of surgery, operation time, and time of exit from the operating room; and outcomes data. Patient age was grouped into five categories: <1 (Infants), 1–3 (Toddlers), 4–5 (Preschoolers), 6–11 (School-age children), and 12–17 years (Adolescents), to reflect key developmental stages in pediatric practice. This categorization allows for the identification of age-specific UHA risks. Operating time was divided into four groups: <0.5, 0.5–1, 1–2, and >2 h, representing typical surgery durations and facilitating the analysis of the impact of longer procedures on UHA risk. Reasons for UHA and any interventions provided were categorized in the following categories by two authors working independently, using frameworks from previous studies [5, 7, 10]: anesthetic (e.g., postoperative nausea and vomiting [PONV]), surgical (e.g., bleeding), medical (e.g., seizure, fever, diarrhea), or social (e.g., inadequate home support). In cases of disagreement between the two authors regarding the categorization of reasons or interventions, they discussed the cases together until a consensus was reached on the final categorization. Additional information was collected on length of hospital stay, subsequent hospital course after admission, discharge status, and mortality.

### Definition of UHA

UHA was defined as any hospital admission requiring an overnight stay following day surgery that was not planned preoperatively. This included patients who were unable to be discharged as planned on the same day as their surgery and also patients who returned to the hospital emergency department and required hospital admission within 24 h of the initial discharge.

### Outcome measures

The primary outcome was the prevalence of UHA, which was defined as the proportion of patients experiencing UHA among the study population. The secondary outcomes were the reasons for UHA, the interventions implemented, the subsequent hospital course in patients who experienced UHA, discharge status, and mortality.

### Statistical analysis

The data were examined for normality of distribution using the Shapiro–Wilk test. Continuous variables are presented as the mean ± standard deviation or as the median (interquartile range) as appropriate. Categorical variables are shown as the number (percentage). Bivariate analysis was performed using Student’s test or the Mann–Whitney *U* test for continuous variables and the chi-squared test or Fisher’s exact test for categorical variables. Risk factors for UHA after pediatric day surgery were identified by logistic regression. Based on the regression analysis assumption of each sample independence, the second or subsequent anesthesia cases in the same personnel during the study period were excluded from applying logistic regression analysis. Potential risk factors were selected based on the previous literature, including age, time of exit from the operating room, and operation time [7]. The operation time variable was incorporated into the logistic regression model as a continuous variable. Meanwhile, a dummy variable was used for the time of exit from operating room to represent the three categories: morning (09:00–11:59), afternoon (12:00–14:59), and evening (after 15:00). Morning exit time (09:00–11:59) was set as the reference category to compare the odds of UHA against the other two time categories. All statistical analyses were performed using STATA 17.0^®^ (StataCorp), with a two-sided *P* value of <0.05 indicating statistical significance.

## Results

### Primary outcome and patient characteristics

**Fig. 1:**
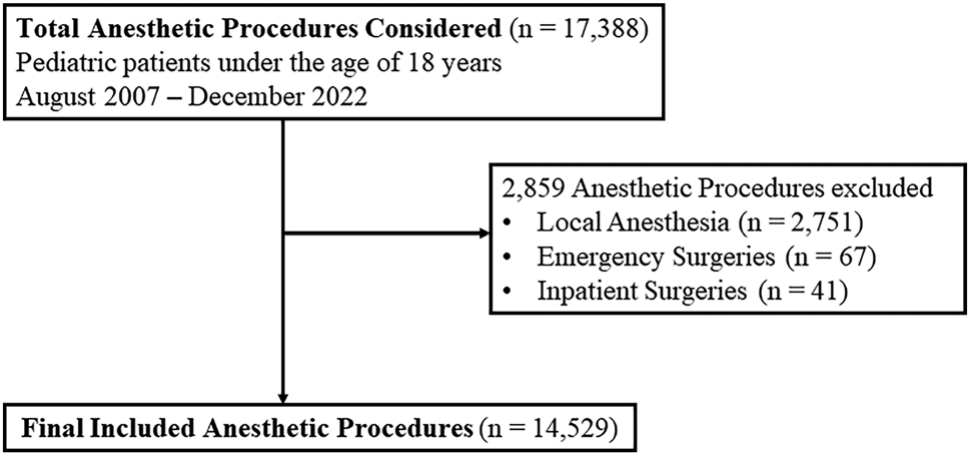
Inclusion diagram

Between August 2007 and December 2022, 14,529 anesthetic procedures for day surgery in 11,818 patients were performed. Twenty-eight (0.19%) of 14,529 anesthetic procedures at our hospital during the study period required UHA. Figure 1 illustrates the inclusion and exclusion process for anesthetic procedures in this study. Table 1 summarizes the characteristics of the 14,529 anesthetic procedures for whom complete data were available. Patients who required UHA were older than those who did not (age group 12–17 years; 18% vs. 4.0%, *P* = 0.045) and were more likely to have a higher ASA-PS score (score > 2; 3.6% vs. 0.11%, *P* = 0.036). Patients who required UHA also underwent more urologic procedures (43% vs. 18%, *P* = 0.017) and were more likely to have surgeries lasting >2 h (3.6% vs. 0.19%, *P* < 0.001) and to exit the operating room after 15:00 (18% vs. 0.34%, *P* < 0.001).

**Table 1:** Characteristics of 14,529 patients according to UHA status

Characteristic	Non-UHA (*n* = 14,501)	UHA (*n* = 28)	*P* value
Sex, *n* (%)			0.252
Male	8733 (60)	20 (71)	
Female	5768 (40)	8 (29)	
Age, years, median (IQR)	4 (2, 6)	4.5 (3, 7.5)	0.16
Age, years, *n* (%)			0.045
<1	1513 (10)	2 (7.1)	
1–3	5277 (36)	10 (36)	
4–5	3140 (22)	5 (18)	
6–11	3993 (28)	6 (21)	
12–17	578 (4.0)	5 (18)	
BMI, median (IQR)	16 (15, 17)	17 (15, 17)	0.484
ASA-PS score, *n* (%)			0.036
1	12,741 (88)	24 (86)	
2	1744 (12)	3 (11)	
3	16 (0.11)	1 (3.6)	
Type of surgery, *n* (%)			0.017
General	4071 (28)	10 (36)	
Otolaryngology	2807 (19)	2 (7.1)	
Urology	2615 (18)	12 (43)	
Plastic	2453 (17)	3 (11)	
Ophthalmology	1532 (11)	0 (0)	
Dental	547 (3.8)	1 (3.6)	
Orthopedic	476 (3.3)	0 (0)	
Operation time, h, *n* (%)			<0.001
<0.5	10,225 (71)	9 (32)	
0.5–1	3562 (25)	10 (36)	
1–2	686 (4.7)	8 (29)	
>2	28 (0.19)	1 (3.6)	
Time of exit from OR, *n* (%)			<0.001
Morning (09:00–11:59)	9585 (66)	12 (43)	
Afternoon (12:00–14:59)	4867 (34)	11 (39)	
Evening (after 15:00)	49 (0.34)	5 (18)	
OSA, *n* (%)			0.534
No symptoms	3350 (23)	7 (25)	
Snoring without apnea	900 (6.2)	3 (11)	
Snoring with apnea	71 (0.49)	0 (0)	

BMI was calculated as kg/m^2^. Data for BMI and OSA were missing for 9161 and 10,198 cases, respectively. Otherwise, no missing values were identified

*ASA-PS* American Society of Anesthesiologists physical status, *BMI* body mass index, *IQR* interquartile range, *OR* operating room, *OSA* obstructive sleep apnea, *UHA* unplanned hospital admission

### Reasons for UHA

Twenty-four (86%) of the 28 patients with UHA required inpatient admission before their initial discharge and 4 (14%) required inpatient admission following a visit to the emergency department within 24 h of day surgery. The most common reasons for UHA were anesthesia-related, the most frequent being PONV, which occurred in 10 patients (36%), and required a mean hospital stay of 2 days (Table 2). Newly identified medical conditions (hypoglycemia, seizure, ascites, gastroenteritis) accounted for 4 UHA (14%) and required the longest average hospital stay at 22 days. Surgical complications led to 6 UHA (22%) that required a mean hospital stay of 2 days (Table 2).

**Table 2:** Reasons for unplanned hospital admissions (*n* = 28)

Reason	*n* = 28	Length of hospital stay (days)
Anesthetic		
Postoperative nausea and vomiting	10 (36)	2 (2, 2)
Inadequate pain control	2 (7.1)	2 (2, 2)
Medical		
Newly identified medical condition	4 (14)	22 (4.5, 109)
Fever	2 (7.1)	2 (2, 2)
Exacerbation of preexisting medical condition	1 (3.6)	2 (2, 2)
Surgical		
Excessive bleeding/surgical complication	3 (11)	2 (2, 6)
More extensive surgery planned/subsequent procedure planned	3 (11)	2 (2, 2)
Social		
Parent request	1 (3.6)	2 (2, 2)
Late exit from operating room (after 15:00)	2 (7.1)	2 (2, 2)

Values are presented as the number (percentage) except for admission days, which are shown as the median (interquartile range)

Among the anesthetic-related admissions, intravenous fluids were the most common intervention (*n* = 10, 36%), followed by antiemetic treatment with medications such as dexamethasone, metoclopramide, and famotidine (*n* = 6, 21%). Analgesic medications were provided in 2 cases (7.1%), including acetaminophen, nonsteroidal anti-inflammatory drugs, and fentanyl.

For medical-related admissions, nonsurgical interventions were predominant (*n* = 6), including intravenous fluids, intensive care for seizures and hypoglycemia, seizure treatment, diuretic therapy for ascites, and inhalation treatment for asthma. Surgical intervention was required in 1 case (3.6%), where endoscopic examination revealed ascites, leading to a diagnosis of Wilms tumor and tumor resection. One other patient (3.6%) was managed with observation only for a high fever. Intravenous line insertion was not feasible, and the patient was supported through breastfeeding (Table 3).

**Table 3:** Interventions provided for unplanned hospital admissions according to reason (*n* = 28)

Reason	*n* (%)	Intervention
Anesthetic		
Intravenous fluids	10 (36)	
Antiemetic	6 (21)	Dexamethasone, metoclopramide, famotidine
Analgesia	2 (7.1)	Acetaminophen, nonsteroidal anti-inflammatory drugs, fentanyl
Observation only	0 (0)	
Medical		
Nonsurgical intervention	6 (21)	Intravenous fluids (2 cases), intensive care for seizures and hypoglycemia, seizure treatment, diuretic for ascites, inhalation treatment for asthma
Surgical intervention	1 (3.6)	Endoscopy revealing ascites, subsequently diagnosed with Wilms tumor and tumor resection performed
Observation only	1 (3.6)	
Surgical		
Surgical intervention	6 (18)	Hemostatic surgery for bleeding (2 cases), abscess drainage for infection, revision surgery for rectovaginal fistula found after vaginoplasty, surgery for a suspected polyp that was found to be hemorrhoids requiring hemorrhoidectomy, planned inguinal hernia repair but suspected cancer found near the testicle
Nonsurgical intervention	1 (3.6)	Antibiotics administration for surgical site infection
Observation only	0 (0)	
Social		
Observation only	3 (11)	

Eight children in the unplanned hospital admission group received two interventions

In the surgical category, all 6 patients (18%) required surgical intervention to address the complications, including 2 cases of hemostatic surgery for bleeding, abscess drainage for infection, revision surgery for rectovaginal fistula found after vaginoplasty, surgery for a suspected polyp that was found to be hemorrhoids requiring hemorrhoidectomy, and a planned inguinal hernia repair where a suspected cancer was found near the testicle. One patient (3.6%) also received nonsurgical intervention with antibiotic therapy for a surgical site infection.

In the social category, observation without specific medical or surgical intervention was undertaken for 3 patients (11%). Eight children in the hospital admission group received more than one type of intervention.

### Subsequent hospital course

Twenty-four (86%) of the 28 patients who experienced UHA were discharged after a hospital stay of 1–2 days. The remaining 4 patients (14%) experienced more extended admissions that ranged from 6 to 182 days. Two patients experienced postoperative infections and recovered after appropriate treatment. One patient returned to the emergency department with fever, diarrhea, and vomiting after inguinal hernia repair and was diagnosed with infectious gastroenteritis. This patient received intravenous fluids and was discharged after a 7-day hospital stay. Another patient developed a postoperative wound infection after inguinal hernia repair, presenting with fever. The infection required incision and drainage, followed by intravenous antibiotic therapy, and the patient was discharged after a 6-day hospital stay. The third patient was hospitalized for a prolonged period after an incidental diagnosis of Wilms tumor during admission for an unrelated condition. This patient underwent surgical resection of the tumor followed by chemoradiotherapy, with a total hospital stay of 182 days. The patient passed away 3 months after discharge due to progression of the underlying cancer. One patient experienced a severe postoperative course, with complications likely associated with critical hypoglycemia, and died during the follow-up period.

### Risk factors for UHA

The results of the multivariable logistic regression analysis of the first anesthetic procedures in 11,818 patients during the study period are summarized in Table 4. The incidence of the UHA was 27 (0.23%). The risk of UHA was significantly higher in patients who exited the operating room after 15:00 than in those who exited between 09:00 and 11:59 (adjusted Odds ratio 29.3; 95% CI [7.09, 121]; *P* < 0.001). Longer operation time was also associated with a higher risk of UHA (adjusted Odds ratio 1.03; 95% CI [1.01, 1.04]; *P* < 0.001).

**Table 4:** Risk factors associated with unplanned hospital admission identified by multivariable logistic regression analysis (*n* = 14,529)

Risk factor	Odds ratio (95% CI)	*P* value
Age, years	1.06 (0.95, 1.17)	0.301
Time of exit from the OR		
Morning (09:00–11:59)	1 (reference)	
Afternoon (12:00–14:59)	1.16 (0.47, 2.84)	0.750
Evening (after 15:00)	30.1 (7.72, 117)	<0.001
Operation time	1.03 (1.02, 1.04)	<0.001
ASA-PS score	1.19 (0.45, 3.18)	0.721

Hosmer–Lemeshow goodness of fit test of the model: *χ*^2^ (8) = 6.16, *P* = 0.629. The VIFs for all covariates were <5 (the highest VIF value was 1.12)

*ASA-PS* American Society of Anesthesiologists physical status, *OR* operating room, *VIF* variance inflation factor

### Subgroup analysis

The median (IQR) operation time for the UHA group was significantly longer than that for the non-UHA group, at 106 (72, 112) min versus 15 (6, 36) min, respectively (*P* = 0.0057). This result supports the finding that surgery duration independently contributes to UHA risk and further validates the multivariable logistic regression analysis in Table 4.

## Discussion

This study investigated the incidence of UHA following pediatric day surgery in a single center in Japan and the related risks, interventions, and subsequent courses for patients. The incidence of UHA following pediatric day surgery was notably low in our study population, with only 28 (0.19%) of 14,529 anesthetic procedures requiring UHA. The risk factors for UHA were exit from the operating room after 15:00 and longer operation time. The most common cause of UHA was anesthesia-related, while the second most prevalent reason was medical. Patients who underwent UHA were typically discharged a few days after admission.

The lower rate of UHA observed in this study compared with previous reports may be related to our institution’s stringent patient selection criteria and comprehensive perioperative care planning. This approach prioritizes patient safety and thorough assessment to ensure that only low-risk patients undergo day surgery, which could contribute to the observed outcomes. In addition, the Japanese healthcare system’s reimbursement structure does not financially incentivize day surgery over inpatient procedures. As a result, our clinical decisions are guided solely by patient safety and medical considerations, without any financial bias. Although this approach may increase social medical costs, it ensures that we select only low-risk patients for day surgery. This prioritization of safety over cost efficiency may contribute to the lower UHA rates observed in our study. In cases where there is any doubt about a patient’s suitability for day surgery, we err on the side of caution and schedule the procedure as an inpatient case.

A further health-economic evaluation study incorporating data such as hospital financial incentives and nationwide medical costs could provide a clearer picture of the risk–benefit balance and financial feasibility of expanding day surgery to include a broader range of procedures and greater complexity of cases. While such data were not available in the present study, conducting such health-economic analysis in future research could offer key insights from the perspective of financial practicability. This may potentially lead to a recalibration of outpatient care models to maintain quality pediatric health care delivery while considering economic factors.

This study addresses a knowledge gap by classifying and evaluating the specific reasons for UHA after pediatric day surgery and the subsequent interventions used. The primary cause for UHA was identified to be PONV, which was typically managed with intravenous fluids and antiemetics. These findings provide valuable insights into the effectiveness of targeted interventions and risk factors associated with UHA. Going forward, we will apply this evidence prospectively to identify patients at greatest risk of UHA before their day surgery procedure. This will allow us to implement preventive strategies and anticipatory management tailored to each patient’s risk profile. Ultimately, this focused analysis will support the refinement and optimization of our pediatric day surgery program by enabling risk-stratified, customized care pathways based on the reason for potential UHA.

In this study, longer operation time and exit time from the operating room after 15:00 were significantly associated with increased risk of UHA after pediatric day surgery, consistent with previous reports [7]. Intriguingly, patient age was not a significant predictor within our cohort. Although there were fewer patients under 1 year of age in the UHA group compared with the non-UHA group, there was a higher proportion of UHA cases among those aged 12–17 years. Although the exact reasons for the increased UHA risk in patients exiting the operating room after 15:00 cannot be determined in this study, one possible explanation is that healthcare providers may feel uncertain about discharging patients due to shorter observation times in the outpatient setting. This could lead to a greater tendency to admit patients after procedures later in the day, regardless of age or procedure duration. The analysis also identified longer operation time as a risk factor, adjusted for age and ASA-PS classification. Therefore, while the timing of procedures may play a role, the higher UHA proportion in the 12–17-year age group does not seem solely attributable to potentially later scheduling. Unfortunately, we could not examine the association between OSA and UHA due to substantial missing data for this variable in our dataset. Future research with standardized data collection for known and potential pediatric risk factors, including OSA, could help formulate evidence-based guidelines tailored to our patient population.

We observed an undesirable postoperative course after day surgery, likely due to critical hypoglycemia. A critical review suggested that prolonged fasting and early discharge without establishing reliable oral intake may have contributed to the postoperative course. In response, we have reinforced adherence to our established discharge criteria and enhanced postoperative monitoring, particularly for medically fragile patients, to ensure adequate feeding and hydration status prior to discharge. This experience underscores the need for meticulous perioperative planning and highlights our commitment to continuous improvement in patient safety.

This study had several limitations that stem from its retrospective observational design. A noticeable gap in the dataset was the absence of comprehensive data on OSA, which precluded a detailed analysis of its impact on UHA. The single-center nature of the research may also limit the generalizability of our findings, and multicenter studies are needed for broader applicability. Furthermore, retrospective collection of data inherently misses some potential risk factors and may be subject to bias owing to non-standardized definitions and reliance on clinical judgment for patient characteristics and comorbidities. This could affect the accuracy and completeness of the data, particularly concerning variables that were not accounted for. In addition, due to the retrospective nature of this study, detailed data on anesthetic agents, vital signs, and airway management techniques were not available. Future prospective research should aim to include these parameters to better understand their association with UHA risk and optimize anesthesia management strategies for pediatric day surgery. In our analysis, there were multiple comparisons between UHA and non-UHA groups that could cause the inflation of type 1 errors. This potentially caused false statistical significance in the results for hypothetical testing between the two groups. Therefore, although our dataset included a relatively large sample size, the statistically significant results must be carefully interpreted. Regarding the variable selection in logistic regression analysis, there can be an over-specification issue in our regression model due to its small outcome number. Therefore, we minimized the number of variables incorporated into the regression model in Table 4. ASA-PS was reported as a potential risk for UHA in day surgery. [7] Since our dataset included 11,808 (99.9%) patients with ASA-PS I or II, we excluded the variable of ASA-PS in our regression models to reduce over-specification. However, ASA-PS possibly remained an unadjusted confounder.

This single-center retrospective study examined epidemiological data on UHA following pediatric day surgery in Japan, analyzing a total of 14,529 anesthetic procedures and revealing a low incidence of 0.19%. The most common reasons for UHA were anesthetic-related factors, particularly PONV, which was managed primarily with intravenous fluids and antiemetic medications. Longer operation time and exit time from the operating room after 15:00 were identified as significant risk factors for UHA. These findings can guide the development of targeted strategies to further reduce UHA and improve the quality of care in pediatric day surgery.

## Prior presentation

Parts of these findings were reported during an invited presentation at the 98th Annual Scientific Meeting of the Korean Society of Anesthesiologists in Busan, Korea on November 6, 2021.

## Data Availability

The anonymized data that support the findings of this study can be provided by the principal investigator upon reasonable request.
